# Aqueous Photoiniferter Polymerization of Acrylonitrile

**DOI:** 10.1021/acsmacrolett.4c00642

**Published:** 2024-11-19

**Authors:** Evan K. Stacy, Mac L. McCormick, Kaden C. Stevens, Penelope E. Jankoski, Jeff Aguinaga, Derek L. Patton, Brent S. Sumerlin, Tristan D. Clemons

**Affiliations:** † School of Polymer Science and Engineering, University of Southern Mississippi, 118 College Drive, Hattiesburg, Mississippi 39402, United States; ‡ George & Josephine Butler Polymer Research Laboratory, Center for Macromolecular Science & Engineering, Department of Chemistry, University of Florida, PO Box 117200, Gainesville, Florida 32611, United States

## Abstract

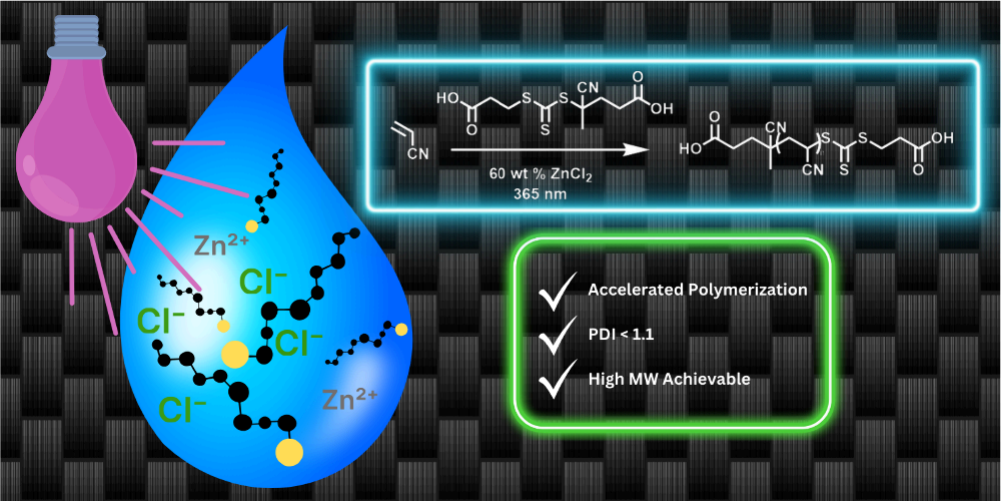

Polyacrylonitrile (PAN) is a key industrial polymer for the production of carbon fiber for high-strength, lightweight composite material applications, with an estimated 90% of the carbon fiber market relying on PAN-based polymers. Traditionally, PAN synthesis is achieved by conventional radical polymerization, resulting in broad molecular weight distributions and the use of toxic organic solvents or surfactants during the synthesis. Additionally, attempts to improve polymer and processing properties by controlled radical polymerization methods suffer from low monomer conversions and struggle to achieve molecular weights suitable for producing high-performance carbon fiber. In this study, we present an aqueous photoiniferter (aqPI) polymerization of acrylonitrile, achieving high monomer conversion and high PAN molecular weights with significantly faster kinetics and dispersity control when compared to traditional methods. This approach allows for the unprecedented control of polymer properties that are integral for downstream processing for enhanced carbon fiber production.

Carbon fiber is undoubtedly the structural material of the 21st century, with its high strength-to-weight ratio making it imperative for the future of weight-sensitive industries including aerospace, automotive, energy, and construction.^1^ Carbon-fiber-reinforced polymers represent the highest performance polymer matrix composites due to the high strength, high modulus, and low density of carbon fibers. Additionally, these composite materials retain high tensile moduli and high strengths at elevated temperatures and extreme environments, offer excellent electrical and thermal conductivity, and display a relatively low coefficient of thermal expansion.^2^ With these benefits, it is anticipated that the global demand for carbon fiber composites will double over the next ten years.^3^ Achieving optimal carbon fiber performance, however, hinges on designing precursor polymers and polymer structures effectively.^4^

Carbon fiber produced from polyacrylonitrile (PAN) dominates the industry, accounting for more than 90% of carbon fiber production.^1,5^ PAN is well suited to forming high-performance carbon fiber due to its ability to form well-defined graphitic structures through a stepwise carbonization process. It is generally accepted that high molecular weights (>150,000 g/mol) of PAN precursor result in enhanced carbon fiber mechanical properties, while narrow polymer dispersity (*Đ* < 1.1) can facilitate improved solution viscosity and processing windows for fiber production.^6,7^ To address this, recent studies have investigated controlled radical polymerization techniques, such as atom transfer radical polymerization (ATRP)^8^ and reversible addition–fragmentation chain transfer (RAFT) polymerization,^6,7,9−13^ for the synthesis of more ideal PAN-based precursor polymers for carbon fiber production. These studies, however, have required long reaction times, involved toxic solvents, and achieved relatively low conversions, yielding poor atom efficiencies. Moreover, acrylonitrile (AN) polymerizations generally struggle to maintain control even at moderate molecular weights, resulting in only a handful of studies achieving relatively low dispersity PAN-based polymers with *M*_n_ > 150 000 g/mol ideal for fiber spinning.^6,7,9,11,14^

An additional challenge for the industrial synthesis of PAN is that PAN is not soluble in its own monomer, rendering bulk polymerizations of AN unfeasible; hence, conventional radical polymerization in solution, suspension, or emulsion is usually employed.^12^ PAN is soluble in polar organic solvents such as *N*,*N*-dimethylformamide (DMF), dimethyl sulfoxide (DMSO), and ethylene carbonate (EC), all of which are currently used for PAN synthesis.^15^ However, these solvents are toxic, expensive, and exhibit high boiling points, making polymer purification at industrial scales difficult.^16,17^ Alternatively, concentrated aqueous solutions of particular inorganic salts, such as sodium thiocyanate (NaSCN, 50 wt %) or zinc chloride (ZnCl_2_, 60 wt %), have been used in carbon fiber processing to dissolve high molecular weight PAN for fiber spinning.^18−20^ The Matyjaszewski laboratory utilized these highly salted aqueous solutions for conventional thermal-initiated RAFT polymerization of AN,^12^ but the results were limited to modest molecular weights (∼60,000 g/mol) that were less than ideal for high-performance carbon fiber production.

Recent work from the Sumerlin laboratory^21^ has demonstrated that aqueous photoiniferter (aqPI) polymerization can generate polymers with ultrahigh molecular weight (UHMW, *M*_n_ greater than 1 × 10^6^ g/mol) and narrow dispersities.^22−26^ The kinetic conditions necessary to achieve UHMW polymers include a high rate of propagation and a low rate of irreversible termination. It has been well-documented that an aqueous reaction medium can support high rates of propagation.^27−29^ Similarly, the photoiniferter approach limits background initiation of low molecular weight chains that lead to irreversible and premature chain termination throughout the polymerization and which are generated continuously during traditional, exogenously initiated RAFT polymerizations.^22^ A recent study by Li et al. explored photoiniferter of AN in EC, achieving high molecular weights, but only at low conversions, again resulting in poor atom economy.^13^ We believe aqPI is an ideal method to achieve rapid, controlled polymerization of high molecular weight PAN at high conversions ideally suited for translation to an industrial setting. In this study, we assess the potential of highly salted aqueous solutions as the polymerization media for aqPI polymerization of AN, for its utility in the development of precursor polymer synthesis for carbon fiber production (Figure 1).

**Figure 1:**
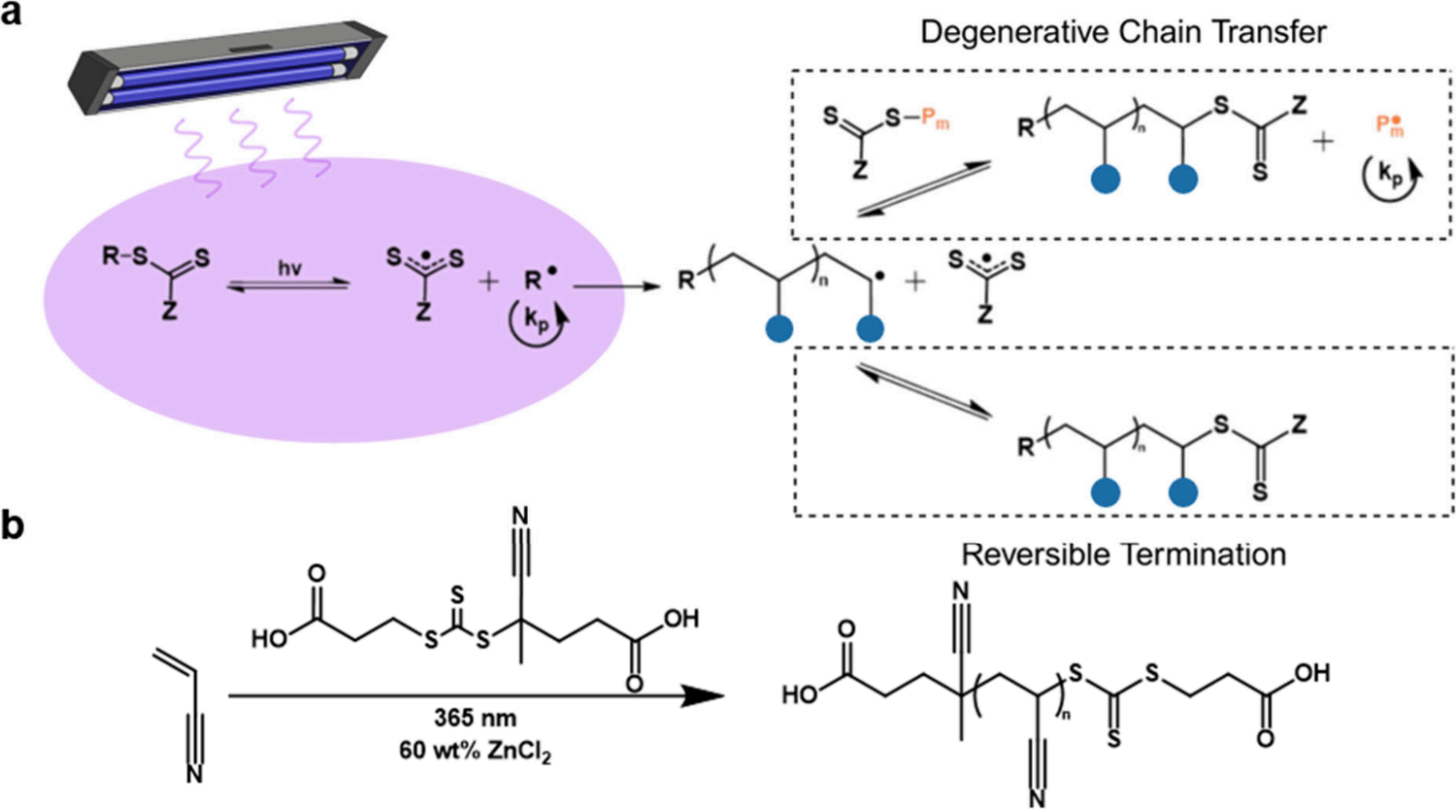
a) Schematic representation of the photoiniferter polymerization process. b) Example reaction conditions explored in this study.

For the aqPI polymerization of AN, 4-((((2-carboxyethyl)thio)carbonothioyl)thio)-4-cyanopentanoic acid (CCPA) was selected as the photoiniferter due to enhanced solubility in the aqueous reaction media resulting from the hydrophilic carboxyl groups (Figure 1b). The nitrile units of the R group were chosen to enhance the rate of initiation/initialization, generating radicals stable enough to favorably cleave upon irradiation or chain transfer while still active enough to initiate polymerization. To assess the utility of aqPI for PAN we initially targeted an AN/CCPA ratio of 500:1 and assessed polymerization kinetics in an aqueous solution of ZnCl_2_ (60 wt %). The reaction yields 90% conversion of AN following 8 h of illumination, as determined from ^1^H NMR spectroscopy (see Supporting Information Figure S1), and follows pseudo-first-order kinetics, with an apparent propagation rate constant of 0.38 h^–1^ (Figure 2a). Additionally, we confirmed 50 wt % aqueous NaSCN was also a suitable polymerization media for AN, with a similar kinetic profile to the ZnCl_2_ yielding an apparent rate constant of 0.37 h^–1^ (Figure 2a). For comparison, the photoiniferter polymerization of AN in EC or DMSO, both common solvents traditionally used for PAN synthesis,^7,9,13,14,30^ resulted in significantly slower apparent rates of propagation of approximately 0.13 and 0.12 h^–1^, respectively (Figure 2a). This enhanced rate of propagation is consistent with previous work that has also observed that aqueous reaction media can support high propagation rates (*k*_p_).^27−29,31^ As expected, under identical aqueous conditions but without the addition of a CTA, uncontrolled polymerization of AN was observed, resulting in what appeared to be a highly branched and cross-linked PAN hydrogel (Figure S2). This UV self-initiation in an aqueous system leading to branching and radical cross-linking is consistent with previous reports,^32−34^ further demonstrating the benefits achieved through appropriate CTA selection to control the photoinitiation, degenerative chain transfer, and reversible termination required for successful photoiniferter polymerization (Figure 1a).

**Figure 2:**
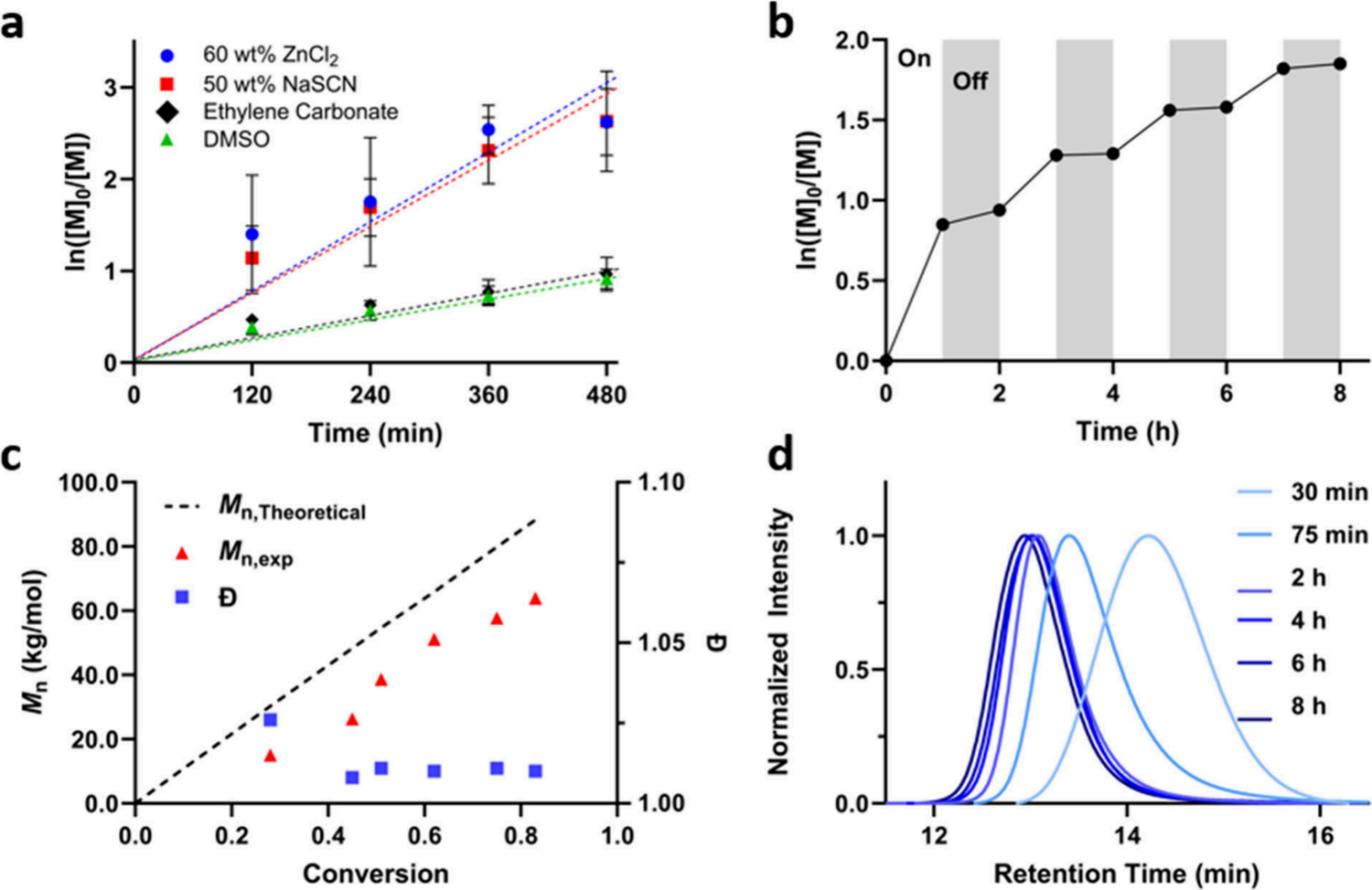
a) AN monomer conversion kinetics with constant illumination of UV light at 27 °C in 60 wt % ZnCl_2_ (blue circles), 50 wt % NaSCN (red squares), EC (black diamonds), and DMSO (green triangles). Data displayed as mean ± standard deviation, *n* = 3 per time point. b) Monomer conversion kinetics with interrupted illumination at 27 °C and 60 wt % ZnCl_2(aq)_ as solvent. c) Theoretical (black) and experimental (red triangles) molecular weights and dispersities (blue squares) of the reaction kinetics as a function of monomer conversion. *M*_n,th_ = [M]_0_/[CCPA]_0_ × MW_M_ × *p* + MW_CCPA_, where [M]_0_, [CCPA]_0_, MW_M_, *p*, and MW_CCPA_ represent initial monomer concentration, initial CCPA concentration, molar mass of the monomer, conversion, and molar mass of CCPA. d) Representative GPC profiles of the purified polymer at several reaction times following polymerization at 27 °C with 60 wt % ZnCl_2(aq)_ as solvent.

The near-linear pseudo-first-order kinetic plots indicate a relatively constant radical concentration is achieved during the photoiniferter process up to high conversions of monomer, suggesting chain-end fidelity is maintained throughout the polymerization. Irreversible decomposition of the thiocarbonylthio end group would decrease radical concentration, reducing the rate of polymerization and producing a negative deviation in the pseudo-first-order kinetic plot.^22^ To further support this point, we investigated the characteristic absorption of CCPA at 310 nm using UV–vis spectroscopy. We observed only a minimal decrease in absorption at 310 nm after 24 h of irradiation at 365 nm, indicating that CCPA resists degradation under the conditions used to perform PAN aqPI polymerization (Figure S3). Photoinitiation allows for temporal control of the polymerization where switching the light source OFF or ON resulted in reversible deactivation or reactivation of the polymerization. Negligible monomer conversion during the period of no irradiation (i.e., light source “OFF”) demonstrates the fast deactivation of the polymerization, in agreement with other photoinitiated polymerization processes.^35^ Finally, we observed strong agreement between theoretical molecular weights (*M*_n,th_) and those observed experimentally by both gel permeation chromatography (GPC, *M*_n,GPC_) and NMR (*M*_n,NMR_) spectroscopy, respectively (Figure 2c and Table S1). The linear increase in *M*_n_ with monomer conversion, coupled with narrow molecular weight distributions (i.e., low *M*_w_/*M*_n_) of the resulting polymer and the symmetrical GPC traces (Figure 2d), indicates control of the polymerization process.

We next investigated the capacity of aqPI to create a high molecular weight PAN optimal for carbon fiber production (Table 1). The accelerated kinetics and absence of exogenous radical during photoiniferter reactions compared to traditional RAFT polymerization have demonstrated the capability to routinely achieve UHMW (*M*_w_ > 1 × 10^6^ g/mol) polymers.^22,36−38^ As a result, we hypothesized that our aqPI conditions could also support the synthesis of the controlled synthesis of high molecular weight polymer suitable for carbon fiber precursor, a feat absent from the literature to the best of our knowledge (entries 6 and 7, Table 1 and Figure 3a). Importantly this is achieved at high monomer conversion and with strong correlation between theoretical and experimental molecular weights, suggesting control and high yield of the polymer product, a challenge observed in previous studies investigating PAN synthesis by RAFT. The samples reached a high level of viscosity during the polymerization, again as evidence of the high molecular weights achieved while maintaining control of the polymerization (Figure 3b). As a demonstration of high chain end fidelity, we were able to synthesize AN diblock copolymers (poly(AN-*b*-AN)). We initially investigated the ability to use high molecular weight PAN of DP 4,000 as a macroCTA by redissolving the purified polymer in 60 wt % ZnCl_2_ and adding 4000 equiv. of monomer to achieve a final DP of approximately 8,000. Interestingly, two distinct linear regions for the kinetics were observed (Figure 3c), with an initial rapid phase of polymerization (0–200 min) followed by an apparent reduction in the rate (200–600 min). We believe this is a result of monomer depletion in the system with the 200 min point of inflection also corresponding to 85% monomer conversion as assessed by proton NMR. A uniform shift in molecular weight was demonstrated by GPC as a further indication of high chain end fidelity of the polymers (Figure S4). Despite a shift toward higher molecular weight, the extent to which these traces overlapped made it difficult to determine the existence of preterminated chains from the initial polymerization of PAN DP 4,000. As such, a lower molecular weight PAN (DP 1,000) sample was also utilized as a macroCTA to synthesize a block copolymer of final DP 5,000. Once again, a uniform shift in molecular weight was demonstrated by GPC, with only a small portion of terminated chains being identified in the lower molecular weight shoulder of the block copolymer (Figure 3d).

**Table 1:** Results of the aqPI Polymerization of AN Conducted in This Study at Ambient Temperatures^a^

Entry	[AN]_0_:[CCPA]_0_	Solvent	[AN]_0_ (mol/L)	Time (h)	Conv.^b^ (%)	*M*_n,th_^c^ (g/mol)	*M*_n,NMR_^d^ (g/mol)	*M*_n,GPC_^e^ (g/mol)	*M*_w_/*M*_n_ (Đ)
1	500:1	60 wt % ZnCl_2_	6.25	8	89	24,300	23,900	29,700	1.02
2	500:1	50 wt % NaSCN	6.25	8	99	25,600	30,000	15,300	1.04
3	500:1	EC	6.25	8	66	17,800	17,000	12,500	1.05
4	500:1	DMSO	6.25	8	60	16,200	18,100	15,100	1.07
5	4,000:1	60 wt % ZnCl_2_	2.00	18	89	189,900	149,400	146,300	1.02
6	9,500:1	60 wt % ZnCl_2_	2.00	18	86	433,400		258,100	1.05
7	20,000:1	60 wt % ZnCl_2_	2.00	18	77	812,200		492,800	1.07
8	25,000:1	60 wt % ZnCl_2_	1.50	24	81	1,072,000		473,500	1.16
9	30,000:1	DMSO	1.25	48	27	434,600		125,300	1.30
10	PAN-*b*-PAN 8,000:1	60 wt % ZnCl_2_	2.00	24	82	287,200		238,500	1.13

a Abbreviations: acrylonitrile (AN), 4-((((2-carboxyethyl)thio)carbonothioyl)thio)-cyanopentanoic acid (CCPA), ethylene carbonate (EC), dimethyl sulfoxide (DMSO).

b Monomer conversion was quantified through ^1^H NMR.

c Calculated according to *M*_n,th_ = ([AN]_0_ × conversion × MW_AN_)/[CCPA]_0_ + MW_CCPA_.

d *M*_n,NMR_ calculated by ^1^H NMR on purified polymer comparing proton signals of CCPA end group relative to the proton adjacent to the nitrile in the polymer backbone.

e Molecular weight measured by Gel Permeation Chromatography Multi-Angle Light Scattering (GPC-MALS) using DMAc with 0.5 M LiCl as eluent.

**Figure 3:**
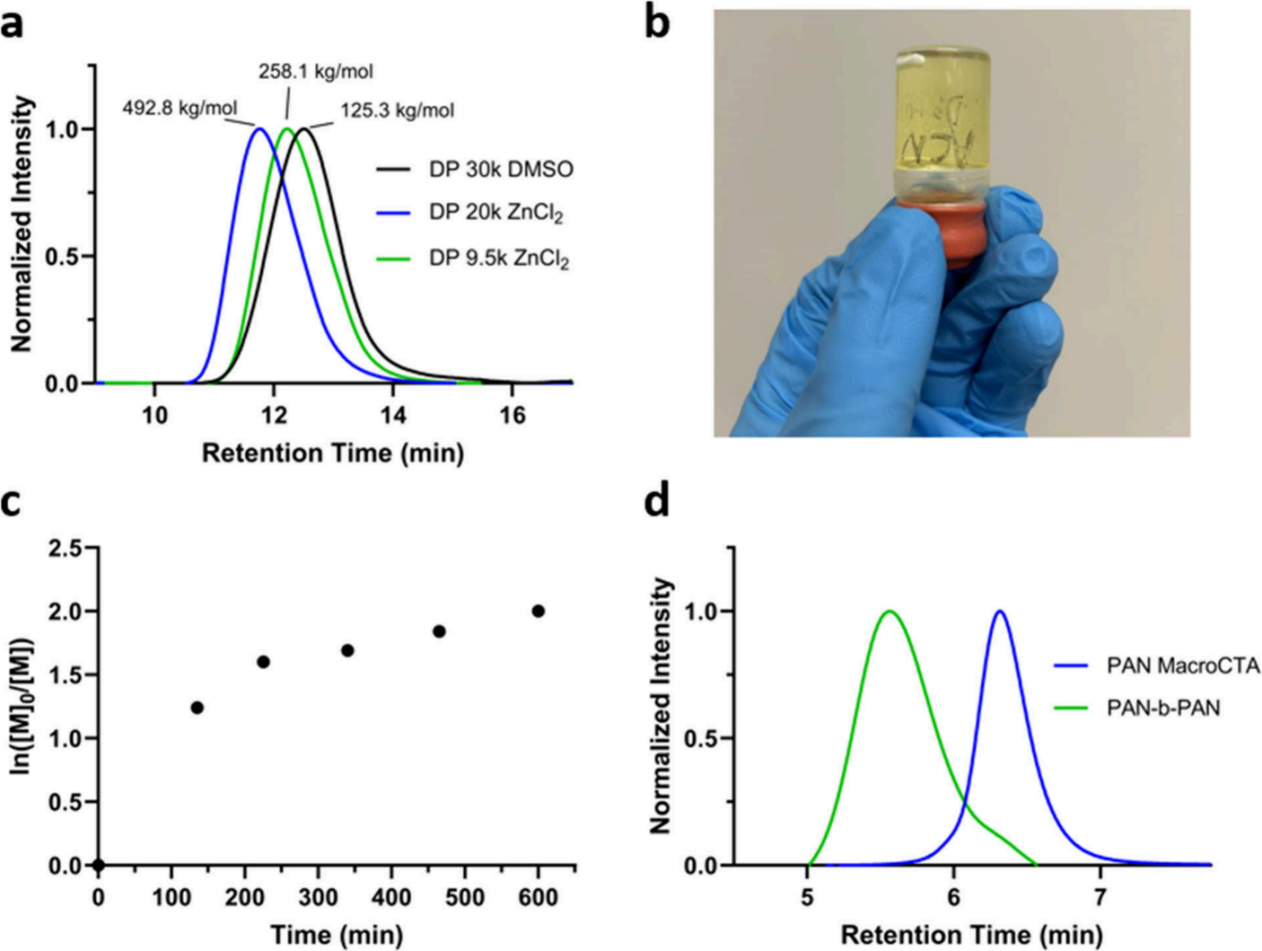
a) GPC traces of high molecular weight PAN prepared by aqPI polymerization with constant illumination of UV light for 24 h, at 27 °C in 60 wt % ZnCl_2_ or DMSO as solvent with target degree of polymerization (DP) provided_._ b) Representative image of PAN DP 9.5k after 12 h of polymerization, demonstrating the high viscosity achieved during polymerization. c) Polymerization kinetics of AN at 27 °C in 60 wt % ZnCl_2_ using PAN DP 4,000 as a macroCTA. d) PAN DP 1,000 macroCTA and chain extended poly(AN-*b*-AN) DP 5,000 GPC traces.

An advantage of photoinitiation is the ability to conduct polymerizations at subambient temperatures. It is hypothesized that, for carbon fiber production, the cyclization yield and formation of “ladder structures” during carbonization of PAN-based precursor polymers could be improved in highly isotactic polymers of acrylonitrile.^39^ Additionally, recent studies have shown that Lewis acids can enhance isotacticity in radical polymerizations.^40−42^ However, achieving high isotacticity in PAN with solution polymerization methods has had limited success.^43^ Okamoto and colleagues demonstrated that temperature significantly influenced tacticity in the radical polymerization of *N*-isopropylacrylamide, with 80% meso diads achieved at 60 °C and over 90% at −20 °C.^42^ Hence we hypothesized that aqPI performed at subambient temperatures and in the presence of Lewis acid additives could enhance tacticity control of PAN.

Initially, we assessed the impact of polymerization kinetics at 8 °C versus 27 °C (i.e., ambient temperatures for the aqPI polymerization). We observed high conversion of monomer despite the subambient temperature investigated, achieving greater than 85% conversion in 8 h of illumination, consistent with observations at ambient temperatures (Figure S5). We then assessed the impact on polymer tacticity by ^13^C NMR spectroscopy, calculating relative amounts of *meso*–*meso* (*mm*), *meso*–*racemo* (*mr*), and *racemo*–*racemo* (*rr*) triads as described previously (Figure S6),^12^ comparing the influence of conducting the polymerizations at 8 °C versus 27 °C. We found that lower temperatures had no significant impact on the tacticity of PAN produced via aqPI in 60 wt % ZnCl_2_ or 50 wt % NaSCN compared to PAN synthesized in traditional organic solvents (Table 2). Additionally, we evaluated aqPI of AN in aqueous Zn(ClO_4_)_2_ due to its demonstrated ability to maintain Lewis acid activity in water (entries 5 and 6, Table 2).^44^ Despite the reduced temperature and presence of these known Lewis acids, no appreciable difference in tacticity was observed. It is likely that even lower temperatures or further optimization of the selected Lewis acid would impart some tacticity control as evidenced by others in the polymerization of acrylamides,^42^ which could further enhance PAN structural properties.

**Table 2:** Influence of Lewis Acid and Reaction Temperature on PAN Tacticity^a^

				C≡N fraction of triads			C—H fraction of triads		
Entry	Solvent	Temp. (°C)	Sample	*mm*	*mr*	*rr*	*mm*	*mr*	*rr*
1	60 wt % ZnCl_2_	28	aqPI (*M*_n_ = 23,861)	0.33	0.50	0.17	0.29	0.50	0.21
2	60 wt % ZnCl_2_	8	aqPI (*M*_n_ = 18,659)	0.26	0.50	0.24	0.30	0.49	0.21
3	50 wt % NaSCN	28	aqPI (*M*_n_ = 30,043)	0.29	0.48	0.23	0.29	0.52	0.19
4	b50 wt % NaSCN	8	aqPI (*M*_n_, N/A)						
5	50 wt % Zn(ClO_4_)_2_	28	aqPI (*M*_n_ = 7,455)	0.33	0.47	0.20	0.29	0.51	0.20
6	50 wt % Zn(ClO_4_)_2_	8	aqPI (*M*_n_ = 7,422)	0.30	0.51	0.19	0.29	0.50	0.21
7	EC	28	PI (*M*_n_ = 17,002)	0.28	0.50	0.22	0.27	0.51	0.22
8	DMSO	28	PI (*M*_n_ = 18,067)	0.26	0.49	0.25	0.27	0.51	0.22

a Abbreviations: aqueous (aq), photoiniferter (PI) polymerization, ethylene carbonate (EC), dimethyl sulfoxide (DMSO).

b Despite observing monomer conversion during polymerization by NMR spectroscopy, NaSCN samples polymerized at 8 °C failed to precipitate, and ^13^C NMR analysis could not be performed.

Interestingly, the reactions conducted at 8 °C in aqueous NaSCN never achieved conversions higher than 70% following 8 h of UV-light exposure. These reactions also failed to produce meaningful quantities of polymer despite significant vinyl bond conversion, as assessed by NMR spectroscopy. We noticed that the evolution of broad, polymeric peaks in the proton NMR spectra did not occur until later time points despite the consumption of vinyl protons early in the reaction (Figure S7). As a result, we hypothesized that there must be a competing reaction occurring between the thiocyanate ions and the acrylonitrile monomer. UV light has been shown to trigger homolytic cleavage of thiocyanogen into thiocyanato radicals that can rapidly react with mono- and disubstituted alkenes to form α,β-dithiocyanates and allylic isothiocyanates at high yields.^45^ Similarly, aqueous solutions of thiocyanate anions can undergo photolysis to form thiocyanate radical anions capable of electron transfer rearrangements.^46,47^ Control experiments conducted in the absence of CTA revealed that vinyl peak consumption occurred rapidly without the evolution of characteristic broad polymer backbone proton signals (Figures S8 and S9). Additionally, the high conversion and large discrepancy observed between the theoretical (*M*_n,th_) and measured (*M*_n,GPC_) molecular weights of the polymer prepared in aqueous NaSCN (Table 1, entry 2) can also be attributed to contributions from this side reaction. This is consistent with the findings of Shimosaka et al., who first demonstrated the ability of NaSCN to act as initiator and sensitizer during AN photopolymerization without any external source of initiating species.^48^ As such, the formation of a weak charge-transfer complex between AN and the thiocyanate anion provides adequate conditions within our system to generate radicals on monomers through electron-transfer-induced rearrangement (Figure S10).^49^ By conducting reactions at equimolar ratios of acrylonitrile and NaSCN, the generation of radical species on the AN monomer resulted in bimolecular termination to form dimers rather than initiating polymerization as witnessed by Shimosaka and co-workers. Taken together, despite the ability to solubilize AN and PAN with 50 wt % NaSCN, this UV-initiated electron-transfer-induced rearrangement significantly impeded molecular weight evolution in our system and as a result is not a suitable solvent for the controlled synthesis of PAN by aqPI. This difference was especially noticeable when compared to the excellent solubility, molecular weight control, and kinetics demonstrated herein with the 60 wt % aqueous ZnCl_2_.

When PAN is processed into carbon fibers, a doping solution is prepared by dissolving the precursor polymer in an organic solvent that is suitable for wet spinning. Importantly, high tensile strength fibers can only be achieved if the precursor polymer possesses a high molecular weight (∼10^5^ g/mol)^7^ and a low dispersity (*Đ* < 2.0) to support dissolution and fiber spinning.^6^ Thus, the polymer characteristics afforded through the aqPI conditions should yield precursors that are highly suitable for manufacturing high-performance carbon fibers. However, residual transition metal residues can be detrimental during carbon fiber production, resulting in void formation and artificially high carbon yields.^50^ In order to gauge the effective removal of zinc, polymers were first precipitated into deionized water and washed three times before lyophilization. Dried PAN powder was then analyzed by X-ray photoelectron spectroscopy (XPS) to quantify residual zinc content (Figure 4). After initial purification in water, the analyzed samples resulted in residual zinc content of 3.60 at. %, which was likely trapped within polymer droplets during precipitation. To avoid this, dried PAN was dissolved in DMSO and reprecipitated in deionized water before further analysis. XPS spectra of the reprecipitated material after one wash cycle yielded zinc content only slightly above the detectable limit (approximately 0.1 atomic %, Figure 4b). The removal of zinc from the final polymer product was also assessed by scanning electron microscopy (SEM) and confirmed by energy dispersive X-ray spectroscopy (EDS) experiments, where zinc following washing was measured to be on the order of 0.01 wt % (Figure S11).

**Figure 4:**
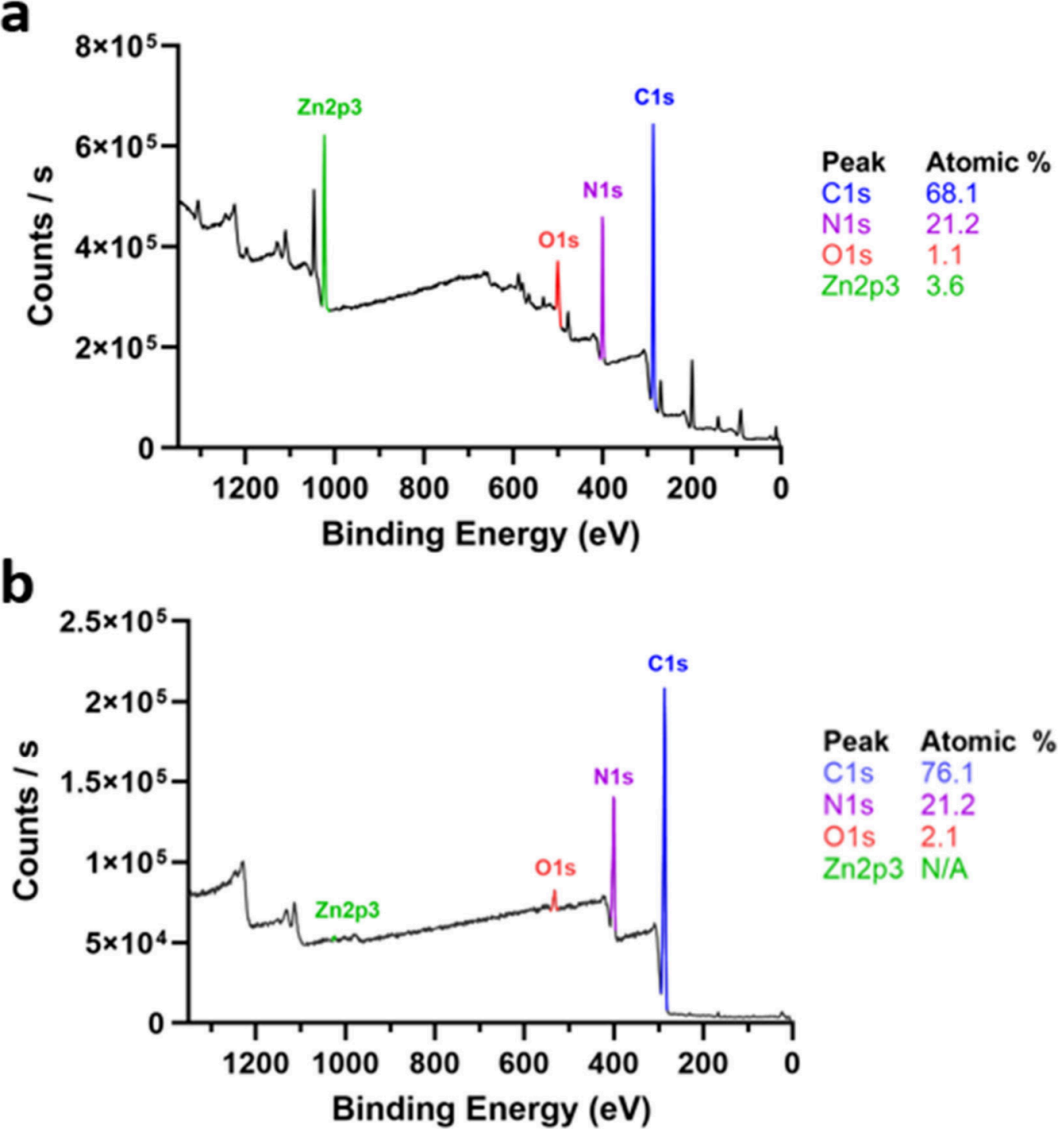
XPS spectra of PAN samples following a) direct precipitation and washing with deionized water and b) redissolution in DMSO and precipitation in deionized water.

In conclusion, we present a facile approach to the controlled synthesis of high molecular weight PAN. To achieve this, we have utilized aqPI, which benefits from rapid propagation and high livingness ideal for achieving ultrahigh molecular weight of PAN. To the best of our knowledge, our aqPI polymerization approach exhibited the fastest propagation rates and highest molecular weights reported for PAN while maintaining low dispersity. The spatiotemporal control of the polymerization along with the ability to prepare diblocks of PAN demonstrate the high degree of chain end fidelity maintained throughout the polymerization process. The use of water as a solvent provides a more environmentally friendly alternative compared to traditional organic solvents used for the industrial synthesis of the PAN precursor polymer currently. We also observed a UV-induced side reaction during the polymerization of AN in the presence of the thiocyanate anion that significantly impeded molecular weight evolution, making this solution less than ideal for aqPI polymerization. Despite the advances in PAN synthesis and control demonstrated herein, there are still limitations in optimizing its structural properties with respect to controlling the polymer tacticity that will be the focus of future studies in this area. Taken together, our approach controls PAN dispersity up to high molecular weights during aqPI polymerization in 60 wt % ZnCl_2_, which could provide advantages when considering the downstream processing of PAN into carbon fiber including lowering viscosity during the wet fiber spinning process and enhancing mass retention through the carbonization process, respectively.

## Supporting Information Available

Additional figures, description of synthetic procedures, and a detailed explanation of materials and methods used (PDF)

## References

[ref1] Le N. D.; Varley R. J.; Hummel M.; Trogen M.; Byrne N. A review of future directions in the development of sustainable carbon fiber from bio-based precursors. Mater. Today Sustain 2022, 20, 100251 10.1016/j.mtsust.2022.100251.

[ref2] Huang X. S. Fabrication and Properties of Carbon Fibers. Materials 2009, 2 (4), 2369–2403. 10.3390/ma2042369.

[ref3] Global Carbon Fiber Reinforced Thermoplastic Composites (CFRTP) Market - Industry Trends and Forecast to 2030. Data Bridge Market Research, 2023. https://www.databridgemarketresearch.com/reports/global-cfrtp-market (accessed January 6, 2024).

[ref4] Chand S. Carbon fibers for composites. J. Mater. Sci. 2000, 35 (6), 1303–1313. 10.1023/A:1004780301489.

[ref5] Baker D. A.; Rials T. G. Recent advances in low-cost carbon fiber manufacture from lignin. J. Appl. Polym. Sci. 2013, 130 (2), 713–728. 10.1002/app.39273.

[ref6] Kaur J.; Millington K.; Cai J. Y. Rheology of polyacrylonitrile-based precursor polymers produced from controlled (RAFT) and conventional polymerization: Its role in solution spinning. J. Appl. Polym. Sci. 2016, 133 (48), 44273 10.1002/app.44273.

[ref7] Cai J. Y.; McDonnell J.; Brackley C.; O’Brien L.; Church J. S.; Millington K.; Smith S.; Phair-Sorensen N. Polyacrylonitrile-based precursors and carbon fibers derived from advanced RAFT technology and conventional methods - The 1st comparative study. Mater. Today Commun. 2016, 9, 22–29. 10.1016/j.mtcomm.2016.09.001.

[ref8] Pan X. C.; Lamson M.; Yan J. J.; Matyjaszewski K. Photoinduced Metal-Free Atom Transfer Radical Polymerization of Acrylonitrile. ACS Macro Lett. 2015, 4 (2), 192–196. 10.1021/mz500834g. 35596430

[ref9] Moskowitz J. D.; Abel B. A.; McCormick C. L.; Wiggins J. S. High Molecular Weight and Low Dispersity Polyacrylonitrile by Low Temperature RAFT Polymerization. J. Polym. Sci. Pol Chem. 2016, 54 (4), 553–562. 10.1002/pola.27806.

[ref10] Moskowitz J. D.; Wiggins J. S. Thermo-oxidative stabilization of polyacrylonitrile and its copolymers: Effect of molecular weight, dispersity, and polymerization pathway. Polym. Degrad. Stab. 2016, 125, 76–86. 10.1016/j.polymdegradstab.2015.12.025.

[ref11] Sayyar S.; Moskowitz J.; Fox B.; Wiggins J.; Wallace G. Wet-spinning and carbonization of graphene/PAN-based fibers: Toward improving the properties of carbon fibers. J. Appl. Polym. Sci. 2019, 136 (36), 47932 10.1002/app.47932.

[ref12] Kopec M.; Krys P.; Yuan R.; Matyjaszewski K. Aqueous RAFT Polymerization of Acrylonitrile. Macromolecules 2016, 49 (16), 5877–5883. 10.1021/acs.macromol.6b01336.

[ref13] Li J. J.; Ding C. L.; Zhang Z. B.; Zhu J.; Zhu X. L. Photo-induced reversible addition-fragmentation chain transfer (RAFT) polymerization of acrylonitrile at ambient temperature: A simple system to obtain high-molecular-weight polyacrylonitrile. React. Funct Polym. 2017, 113, 1–5. 10.1016/j.reactfunctpolym.2017.02.003.

[ref14] Moskowitz J.; Wiggins J. Thermo-oxidative stabilization of polyacrylonitrile and its copolymers: Effect of molecular weight, dispersity, and polymerization pathway. Polym. Degrad. Stab. 2016, 125, 76–86. 10.1016/j.polymdegradstab.2015.12.025.

[ref15] Iovleva M. M.; Smirnova V. N.; Budnitskii G. A. The solubility of polyacrylonitrile. Fibre Chem. 2001, 33 (4), 262–264. 10.1023/A:1012934313303.

[ref16] Morosoff N.; Stannett V. Some effects of small amounts of residual solvent on polyacrylonitrile film. Journal of Macromolecular Science, Part B 1980, 17 (1), 157–161. 10.1080/00222348008212805.

[ref17] Kim D.; Moreno N.; Nunes S. P. Fabrication of polyacrylonitrile hollow fiber membranes from ionic liquid solutions. Polym. Chem-Uk 2016, 7 (1), 113–124. 10.1039/C5PY01344E.

[ref18] Serkov A. T.; Budnitskii G. A. Mechanism of polyacrylonitrile fibre spinning by the thiocyanate method. Fibre Chem. 1994, 25 (5), 335–341. 10.1007/BF00551621.

[ref19] Nam C. W.; Kim Y. H.; Ko S. W. Blend fibers of polyacrylonitrile and water-soluble chitosan derivative prepared from sodium thiocyanate solution. J. Appl. Polym. Sci. 2001, 82 (7), 1620–1629. 10.1002/app.2001.

[ref20] Bajaj P.; Roopanwal A. K. Thermal stabilization of acrylic precursors for the production of carbon fibers: An overview. J. Macromol. Sci. R M C 1997, C37 (1), 97–147. 10.1080/15321799708014734.

[ref21] Hughes R. W.; Lott M. E.; Olson S R. A.; Sumerlin B. S. Photoiniferter polymerization: Illuminating the history, ascendency, and renaissance. Prog. Polym. Sci. 2024, 156, 101871 10.1016/j.progpolymsci.2024.101871.

[ref22] Carmean R. N.; Becker T. E.; Sims M. B.; Sumerlin B. S. Ultra-High Molecular Weights via Aqueous Reversible-Deactivation Radical Polymerization. Chem-Us 2017, 2 (1), 93–101. 10.1016/j.chempr.2016.12.007.

[ref23] Lott M. E.; Trachsel L.; Schué E.; Davidson C. L. G. I. V.; Olson S R. A.; Pedro D. I.; Chang F.; Hong Y.; Sawyer W. G.; Sumerlin B. S. Ultrahigh-Molecular-Weight Triblock Copolymers via Inverse Miniemulsion Photoiniferter Polymerization. Macromolecules 2024, 57 (9), 4007–4015. 10.1021/acs.macromol.4c00366.

[ref24] Diodati L. E.; Wong A. J.; Lott M. E.; Carter A. G.; Sumerlin B. S. Unraveling the Properties of Ultrahigh Molecular Weight Polyacrylates. Acs Appl. Polym. Mater. 2023, 5 (12), 9714–9720. 10.1021/acsapm.3c02191.

[ref25] Davidson C. L. G. I. V.; Lott M. E.; Trachsel L.; Wong A. J.; Olson R. A.; Pedro D. I.; Sawyer W. G.; Sumerlin B. S. Inverse Miniemulsion Enables the Continuous-Flow Synthesis of Controlled Ultra-High Molecular Weight Polymers. ACS Macro Lett. 2023, 12 (9), 1224–1230. 10.1021/acsmacrolett.3c00431. 37624643

[ref26] Olson R. A.; Lott M. E.; Garrison J. B.; Davidson C. L. G. I. V.; Trachsel L.; Pedro D. I.; Sawyer W. G.; Sumerlin B. S. Inverse Miniemulsion Photoiniferter Polymerization for the Synthesis of Ultrahigh Molecular Weight Polymers. Macromolecules 2022, 55 (19), 8451–8460. 10.1021/acs.macromol.2c01239.

[ref27] Read E.; Guinaudeau A.; Wilson D. J.; Cadix A.; Violleau F.; Destarac M. Low temperature RAFT/MADIX gel polymerisation: access to controlled ultra-high molar mass polyacrylamides. Polym. Chem-Uk 2014, 5 (7), 2202–2207. 10.1039/c3py01750h.

[ref28] Lowe A. B.; McCormick C. L. Aqueous RAFT polymerization: Recent developments in synthesis of functional water-soluble (Co)polymers with controlled structures. Acc. Chem. Res. 2004, 37 (5), 312–325. 10.1021/ar0302484. 15147172

[ref29] Lowe A. B.; McCormick C. L. Reversible addition-fragmentation chain transfer (RAFT) radical polymerization and the synthesis of water-soluble (co)polymers under homogeneous conditions in organic and aqueous media. Prog. Polym. Sci. 2007, 32 (3), 283–351. 10.1016/j.progpolymsci.2006.11.003.

[ref30] Moskowitz J. D.; Wiggins J. S. Semibatch RAFT copolymerization of acrylonitrile and N-isopropylacrylamide: Effect of comonomer distribution on cyclization and thermal stability. Polymer 2016, 84, 311–318. 10.1016/j.polymer.2015.12.035.

[ref31] Fortenberry A. W.; Jankoski P. E.; Stacy E. K.; McCormick C. L.; Smith A. E.; Clemons T. D. A Perspective on the History and Current Opportunities of Aqueous RAFT Polymerization. Macromol. Rapid Commun. 2022, 43, 2200414 10.1002/marc.202200414. PMC10697073 35822936

[ref32] Yan W. Q.; de la Vega J.; Eroglu O.; Heisenberg L.; Wang D. Y. High Power Sunlight-Simulated UV-Induced Radical Polymerization: Self-Initiation and Self-Crosslinking. Macromol. Mater. Eng. 2024, 309 (5), 2300456 10.1002/mame.202300456.

[ref33] Thomas W. M. Mechanism of acrylonitrile polymerization; Springer: Berlin, Heidelberg, 1961; pp 401–441.

[ref34] Mah S.; Park S.; Nam H.; Seoul C. Photopolymerization of acrylonitrile in concentrated aqueous zinc halide solutions. J. Appl. Polym. Sci. 2000, 77 (12), 2588–2594. 10.1002/1097-4628(20000919)77:12<2588::AID-APP50>3.0.CO;2-D.

[ref35] McClelland K. P.; Clemons T. D.; Stupp S. I.; Weiss E. A. Semiconductor Quantum Dots Are Efficient and Recyclable Photocatalysts for Aqueous PET-RAFT Polymerization. ACS Macro Lett. 2020, 9 (1), 7–13. 10.1021/acsmacrolett.9b00891. 35638658

[ref36] Trachsel L.; Stewart K. A.; Konar D.; Hillman J. D.; Moerschel J. A.; Sumerlin B. S. β-Triketones as Reactive Handles for Polymer Diversification via Dynamic Catalyst-Free Diketoenamine Click Chemistry. J. Am. Chem. Soc. 2024, 146 (23), 16257–16267. 10.1021/jacs.4c04664. 38832509

[ref37] Trachsel L.; Konar D.; Hillman J. D.; Davidson C. L. G. I. V.; Sumerlin B. S. Diversification of Acrylamide Polymers via Direct Transamidation of Unactivated Tertiary Amides. J. Am. Chem. Soc. 2024, 146 (2), 1627–1634. 10.1021/jacs.3c12174. 38189246

[ref38] Rho J. Y.; Korpusik A. B.; Hoteit M.; Garrison J. B.; Sumerlin B. S. Ultra-high molecular weight complex coacervates via polymerization-induced electrostatic self-assembly. Polym. Chem. 2024, 15 (18), 1821–1825. 10.1039/D4PY00273C.

[ref39] Wang Y. S.; Xu L. H.; Wang M. Z.; Pang W. M.; Ge X. W. Structural Identification of Polyacrylonitrile during Thermal Treatment by Selective C-13 Labeling and Solid-State C-13 NMR Spectroscopy. Macromolecules 2014, 47 (12), 3901–3908. 10.1021/ma500727n.

[ref40] Li N.; Ding D. D.; Pan X. Q.; Zhang Z. B.; Zhu J.; Boyer C.; Zhu X. L. Temperature programed photo-induced RAFT polymerization of stereo-block copolymers of poly(vinyl acetate). Polym. Chem-Uk 2017, 8 (39), 6024–6027. 10.1039/C7PY01531C.

[ref41] Sun Y.; Fu L. Y.; Olszewski M.; Matyjaszewski K. ATRP of N-Hydroxyethyl Acrylamide in the Presence of Lewis Acids: Control of Tacticity, Molecular Weight, and Architecture. Macromol. Rapid Commun. 2019, 40 (10), 1800877 10.1002/marc.201800877. 30650236

[ref42] Isobe Y.; Fujioka D.; Habaue S.; Okamoto Y. Efficient Lewis acid-catalyzed stereocontrolled radical polymerization of acrylamides. J. Am. Chem. Soc. 2001, 123 (29), 7180–7181. 10.1021/ja015888l. 11459507

[ref43] Jiang J. G.; Lu X. Y.; Lu Y. Preparation of Polyacrylonitrile with Improved Isotacticity and Low Polydispersity. J. Appl. Polym. Sci. 2010, 116 (5), 2610–2616. 10.1002/app.31650.

[ref44] Kobayashi S.; Nagayama S.; Busujima T. Lewis acid catalysts stable in water. Correlation between catalytic activity in water and hydrolysis constants and exchange rate constants for substitution of inner-sphere water ligands. J. Am. Chem. Soc. 1998, 120 (32), 8287–8288. 10.1021/ja980715q.

[ref45] Guy R. G.; Thompson J. J. Pseudohalogen chemistry—VI11Part V: R.G. Guy and I. Pearson, Bull. Chem. Soc. Japan 50, 541 (1977).: Homolytic thiocyanation of mono- and di-substituted alkenes using thiocyanogen and ultraviolet light. Tetrahedron 1978, 34 (5), 541–546. 10.1016/0040-4020(78)80049-0.

[ref46] Dogliotti L.; Hayon E. Flash photolysis study of sulfite, thiocyanate, and thiosulfate ions in solution. J. Phys. Chem. 1968, 72 (5), 1800–1807. 10.1021/j100851a073.

[ref47] Wakamatsu K.; Dairiki J.; Etoh T.; Yamamoto H.; Yamamoto S.; Shigetomi Y. Electron-transfer induced rearrangement of thiocyanate to isothiocyanate. Tetrahedron Lett. 2000, 41 (3), 365–369. 10.1016/S0040-4039(99)02063-8.

[ref48] Shimosaka Y.; Uno Y. Sensitizing Effect of Sodium Thiocyanate on Photo-polymerization of Acrylonitrile. KOBUNSHI RONBUNSHU 1982, 39 (12), 783–790. 10.1295/koron.39.783.

[ref49] Mao T. J.; Eldred R. J. Photopolymerization initiated by triphenylphosphine. Journal of Polymer Science Part A-1: Polymer Chemistry 1967, 5 (7), 1741–1751. 10.1002/pol.1967.150050722.

[ref50] Kaur J.; Millington K.; Smith S. Producing high-quality precursor polymer and fibers to achieve theoretical strength in carbon fibers: A review. J. Appl. Polym. Sci. 2016, 133 (38), 43963 10.1002/app.43963.

