# Supplementary material for: Aqueous Photoiniferter Polymerization of Acrylonitrile
Source: ACS Macro Lett. 2024 Nov 19;13(12):1662–9. doi: 10.1021/acsmacrolett.4c00642 (PMC11656713; doi:10.1021/acsmacrolett.4c00642)
Supplement: Supplementary file 1 — mz4c00642_si_001.pdf [file mz4c00642_si_001.pdf]

Supporting Information

Aqueous Photoiniferter Polymerization of Acrylonitrile

Evan K. Stacy,[1] Mac L. McCormick,[1] Kaden Stevens,[2] Penelope E. Jankoski,[1] Jeff Aguinaga,[1] Derek L. Patton,[1] Brent Sumerlin,[2] and Tristan D. Clemons.[1*]

1 School of Polymer Science and Engineering, University of Southern Mississippi, 118 College Drive, Hattiesburg, MS, 39402, USA.

2 George & Josephine Butler Polymer Research Laboratory, Center for Macromolecular Science & Engineering, Department of Chemistry, University of Florida, PO Box 117200, Gainesville, Florida 32611, USA.

*Correspondence should be addressed to T.D.C (email: Tristan.clemons@usm.edu)

Supporting Information

Table of contents

1 Materials and Methods 2
  1.1 Reagents 2
  1.2 Aqueous Photoiniferter Polymerization of Acrylonitrile 2
  1.3 Aqueous Photoiniferter Polymerization of poly(acrylonitrile-b-acrylonitrile) 3
  1.4 Photoiniferter Polymerization of Acrylonitrile in DMSO 3
  1.5 Photoiniferter Polymerization of Acrylonitrile in Ethylene Carbonate 3
2 Characterization 4
  2.1 Nuclear Magnetic Resonance (NMR) Spectroscopy 4
  2.2 Gel Permeation Chromatography Multi-Angle Light Scattering (GPC-MALS) 4
  2.3 UV-Vis Spectroscopy 4
  2.4 X-Ray Photoelectron Spectroscopy (XPS) 4
  2.5 Scanning Electron Microscopy (SEM) 5
3 Supporting Figures 5
  Figure S1. NMR spectroscopy of acrylonitrile (AN) conversion in aqPI polymerization. 5
  Figure S2. UV initiated aqueous free radical polymerization of acrylonitrile. 6
  Figure S3. Assessment of chain transfer agent photolysis. 7

Figure S4. GPC traces of PAN DP 4,000 macroCTA and chain extended PAN-b-PAN DP 8000........ 7
Figure S5. Kinetic plots of aqPI polymerizations conducted at low temperatures........ 8
Figure S6. Polymer tacticity investigation by ^{13}C NMR........ 9
Figure S7. Kinetics study of polyacrylonitrile DP 500 synthesized in 50 wt% NaSCN at 8 and 27 °C........ 10
Figure S8. ^{1}H NMR of photo mediated acrylonitrile and thiocyanate interaction........ 11
Figure S9. ^{13}C NMR of photo mediated acrylonitrile and thiocyonate interaction........ 12
Figure S10. Schematic representation of UV light catalyzed photolysis of aqueous thiocyanate to form radical anions........ 13
Figure S11. Scanning electron microscopy (SEM) assessment following polymer purification........ 14
4 Supporting Tables........ 15
4.1 Table S1. Polymerization kinetics of acrylonitrile DP 2000 with aliquots taken for GPC analysis to confirm agreement between theoretical and experimental M_n........ 15
5 Supporting Information References........ 15

1 Materials and Methods

1.1 Reagents

Acrylonitrile (AN, Acros Organics, >99%) was passed through a column of inhibitor removers and stored at 4 °C prior to polymerization. Zinc chloride ($ZnCl_2$, Sigma Aldrich, >98%), sodium thiocyanate (NaSCN, Sigma Aldrich, >98%), 4-((((2-carboxyethyl)thio)carbonothioyl)thio)-cyanopentanoic acid (CCPA, Boron Molecular, >99%), dimethyl sulfoxide (DMSO, Sigma Aldrich, >99%), *N,N*-dimethylformamide (DMF, Fisher Chemical, >99%), DMSO-d_6 (Millipore Sigma, >99%), and D_2O (TCI Chemicals, >98%) were used as received. Type II Milli-Q water (Millipore) was used in all experiments.

1.2 Aqueous Photoiniferter Polymerization of Acrylonitrile

In a typical procedure, 20 mg of CCPA (0.065 mmol, 1 equiv), 1.73 g of acrylonitrile (32.5 mmol, 500 equiv), 5 mL of an aqueous solution of $ZnCl_2$ (60 wt%) or NaSCN (50 wt%), and 0.2 mL of DMF (NMR standard) were added to a 10 mL scintillation vial equipped with a stir bar and rubber septa followed by 30 minutes of degassing by bubbling nitrogen. Reactions were then exposed to UV light irradiation (365nm) with magnetic stirring for 8 hours at 8°C or 27°C. The polymer was isolated by precipitation of polymerization solution into excess deionized water and centrifuged. The precipitate was filtered, collected, and lyophilized overnight. Polymerization kinetics were assessed with proton NMR by collecting aliquots from the reaction at relevant time points using a

needle purged with nitrogen gas. Linear regression analysis of polymerizations conducted in triplicate yielded an equation of y = 0.006389x for 60 wt% $ZnCl_2$. Linear regression analysis of polymerizations conducted in triplicate yielded an equation of y = 0.006103x for 50 wt% NaSCN. The apparent propagation rate constant for polymerizations, k^{app}_p was taken as the slope of the linear regression (multiplied by 60 to report as h^{-1}).

1.3 Aqueous Photoiniferter Polymerization of poly(acrylonitrile-b-acrylonitrile)

In a typical procedure, 4.67 mg of CCPA (0.015 mmol, 1 equiv), 0.81 g of acrylonitrile (15.2 mmol, 1,000 equiv), 3.6 mL of an aqueous solution of $ZnCl_2$ (60 wt%), and 0.2 mL of DMF (NMR standard) were added to a 10 mL scintillation vial equipped with a stir bar and rubber septa followed by 30 minutes of degassing by bubbling nitrogen. Reactions were then exposed to UV light irradiation (365 nm) with magnetic stirring for 12 hours at 27°C, before being exposed to air. The polymer was isolated by precipitation of polymerization solution into excess deionized water and centrifuged. The precipitate was filtered, collected, and lyophilized overnight. This polymer was then chain extended with acrylonitrile by adding 199 mg of the PAN macroCTA (0.005 mmol, 1 equiv), 1.08 g of acrylonitrile (20.4 mmol, 4,000 equiv), 6.5 mL of a 60 wt% $ZnCl_2$, and 0.2 mL of DMF (NMR standard) to a 10 mL scintillation vial equipped with a stir bar and rubber septa followed by 30 minutes of degassing by bubbling nitrogen. The chain extension reactions were then exposed to UV light irradiation (365 nm) with magnetic stirring for 12 hours at 27°C, before being exposed to air. Block copolymers were isolated by precipitation into excess deionized water and centrifuged. The precipitate was filtered, collected, and lyophilized overnight.

1.4 Photoiniferter Polymerization of Acrylonitrile in DMSO

In a typical procedure, 20 mg of CCPA (0.065 mmol, 1 equiv), 1.73 g of acrylonitrile (32.5 mmol, 500 equiv), 5 mL DMSO, and 0.2 mL of DMF (NMR standard) were added to a 10 mL scintillation vial equipped with a stir bar and rubber septa followed by 30 minutes of degassing by bubbling nitrogen. Reactions were then exposed to UV light irradiation with magnetic stirring for 8 hours at 27°C. The polymer was isolated by precipitation of polymerization solution into excess deionized water and centrifuged. The precipitate was filtered, collected, and lyophilized overnight. Linear regression analysis of polymerizations conducted in triplicate yielded an equation of y = 0.002039x. The apparent propagation rate constant for polymerizations, k^{app}_p was taken as the slope of the linear regression (multiplied by 60 to report as h^{-1}).

1.5 Photoiniferter Polymerization of Acrylonitrile in Ethylene Carbonate

In a typical procedure, 30 mg of CCPA (0.098 mmol, 1 equiv), 2.59 g of acrylonitrile (48.8 mmol, 500 equiv), 9.92 g of ethylene carbonate (0.113 mol), and 0.2 mL of DMF (NMR standard) were added to a 10 mL scintillation vial equipped with a stir bar and rubber septa followed by 30 minutes of degassing by bubbling nitrogen. Reactions were then exposed to UV light irradiation with

magnetic stirring for 8 hours at 27°C. The polymer was isolated by precipitation of polymerization solution into excess deionized water and centrifuged. The precipitate was filtered, collected, and lyophilized overnight. Linear regression analysis of polymerizations conducted in triplicate yielded an equation of y = 0.002195x. The apparent propagation rate constant for polymerizations, k^{app}_p was taken as the slope of the linear regression (multiplied by 60 to report as h^{-1}).

2 Characterization

2.1 Nuclear Magnetic Resonance (NMR) Spectroscopy

^1H and ^{13}C NMR measurements were performed on a Bruker Avance 400 or 600 MHz spectrometer in an appropriate solvent for the polymerization media (D_2O with 60 wt% $ZnCl_2$, D_2O with 50 wt% NaSCN, or DMSO-d_6). Acquired spectra were analyzed to quantify conversion of monomer (p), predicted molecular weights ($M_{n,theor}$), resulting molecular weights of isolated polymer ($M_{n,NMR}$), and tacticity of PAN homopolymer.

2.2 Gel Permeation Chromatography Multi-Angle Light Scattering (GPC-MALS)

Molecular weights ($M_{n,GPC}$) and molecular weight distributions (M_w/M_n) were determined by gel permeation chromatography multi-angle light scattering (GPC-MALS). The samples were lyophilized to remove water before dissolving 3-5 mg of polymer in 1 ml of DMAc with 50 mM LiCl overnight at room temperature before measurement. Measurements were conducted in DMAc containing 50 mM LiCl as the eluent at a flow rate of 1 mL/min at 50 °C on an Agilent pump system connected to a Wyatt Optilab T-rEX refractive index detector and a Wyatt miniDAWN Treos light scattering detector. Two ViscoGel I-series mixed bed columns with a molecular weight range of 0-20 x 10^3 g/mol and 0-100 x 10^5 g/mol along with a 5 µm guard column were used. Apparent molecular weights were calculated via MALS using reported d*n*/d*c* values.[1]

2.3 UV-Vis Spectroscopy

A typical $ZnCl_2$ polymerization mixture was irradiated with UV light for 24 hours, with aliquots taken at t = 2, 4, 6, 8, and 24 hours. Reaction aliquots were diluted to a CTA concentration of 0.1 mg/mL and UV-Vis measurements were conducted on an Agilent BioTek Synergy H1 microplate reader. Measurements consisted of spectral scans across the range of 290 – 450 nm with acquisitions in 2 nm increments. The degree of photolysis was determined by comparing the measured A_{max} at 310 nm, which is characteristic of the trithiocarbonate.

2.4 X-Ray Photoelectron Spectroscopy (XPS)

Polyacrylonitrile synthesized by aqPI in 60 wt% $ZnCl_2$ was directly precipitated with deionized water, redissolved in DMSO at approximately 10 wt% and then reprecipitated again with deionized

water. Polymer was then dried and crushed with mortar and pestle for assessment by XPS on a Thermo-Fisher ESCALAB Xi+ spectrometer and monochromatic Al K alpha X-ray source 1486.6 eV. 2 spots per sample were assessed with a 650µm spot size. Scans were collected using standard charge compensation mode (e- flood gun) and Ar as source for the flood gun with survey scans initially conducted (pass energy = 150eV, 2 scans, 50ms dwell time, 1.0eV resolution) followed by high resolution scans (pass energy = 20eV, 5 scans, 50ms dwell time, 0.1eV resolution) with a takeoff angle of 90°.

2.5 Scanning Electron Microscopy (SEM)

Polyacrylonitrile synthesized by aqPI in 60 wt% $ZnCl_2$ was directly precipitated with deionized water, redissolved in DMSO at approximately 10 wt%, and then reprecipitated again with deionized water. The polymer was then dried and crushed with mortar and pestle for assessment by SEM on a Zeiss Sigma VP field-emission SEM with Thermo System 7 EDS X-ray detector. Crushed PAN before and after washing were carefully placed on conductive tape and mounted on a SEM stub.

3 Supporting Figures

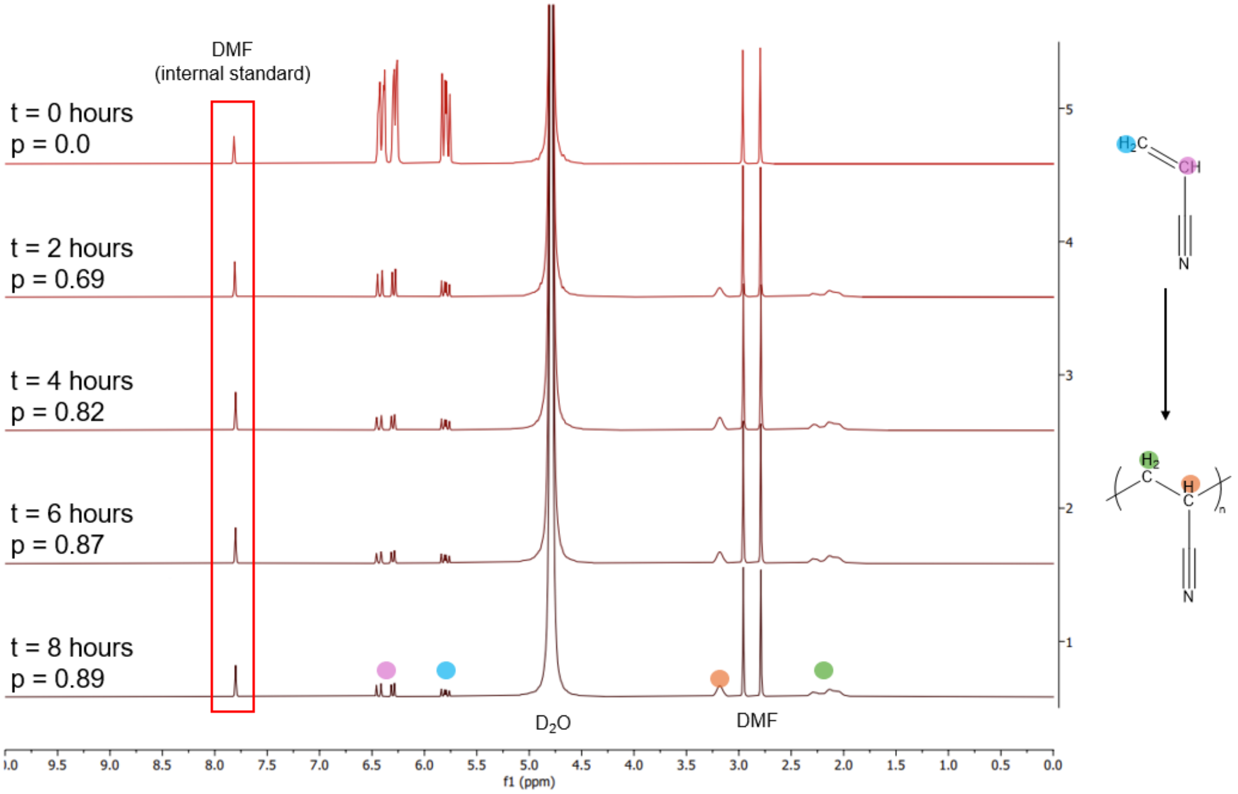

Figure S1. NMR spectroscopy of acrylonitrile (AN) conversion in aqPI polymerization. Representative NMR spectra from an aqPI polymerization of AN in 60 wt% $ZnCl_2$ at t = 0, 2, 4, 6, and 8 hours. To determine conversion, the DMF proton signal at 7.79 ppm

was set to an integration value of 1.00 in each spectra before determining relative integration values of vinyl protons on acrylonitrile monomer between 6.25 and 6.50 ppm. Conversion at a given aliquot (i.e. timepoint), was calculated by the equation ([Integration 6.25-6.50]$_0$ - [Integration 6.25-6.50]$_t$) / [Integration 6.25-6.50]$_0$.

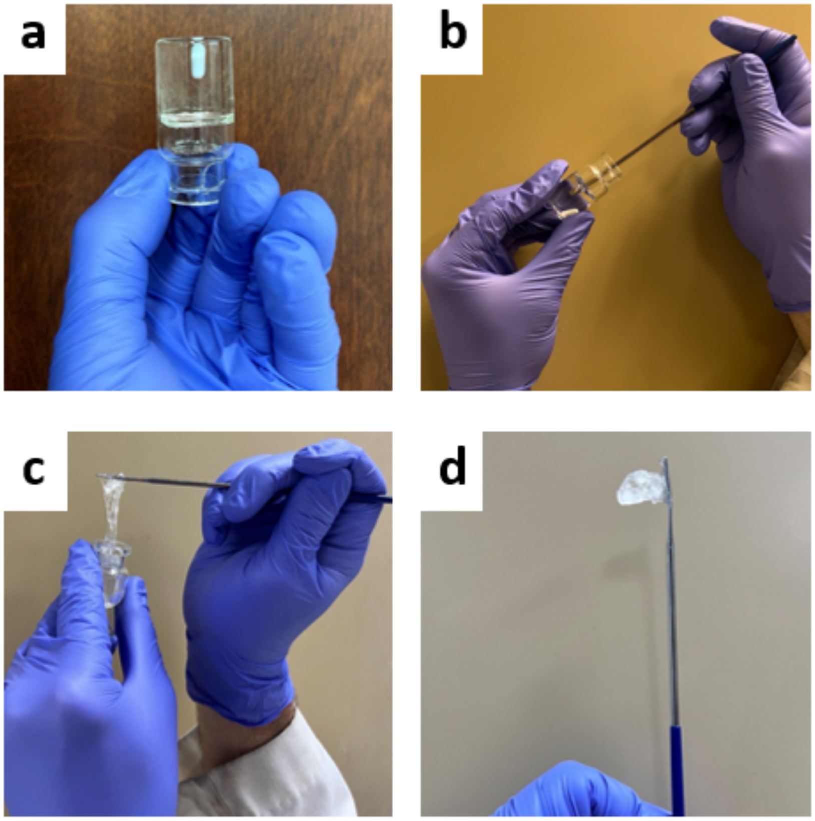

Figure S2. UV initiated aqueous free radical polymerization of acrylonitrile. Samples were prepared under identical conditions as described in Supporting information section 1.1 and Figure 1a with the omission of CTA addition (60 wt% $ZnCl_2$, t=8 hours illumination). Images of the sample following polymerization resulted in a rubbery hydrogel (a and b) capable of c) stretching and d) weight support suggesting significant branching and/or crosslinking occurring during the polymerization.

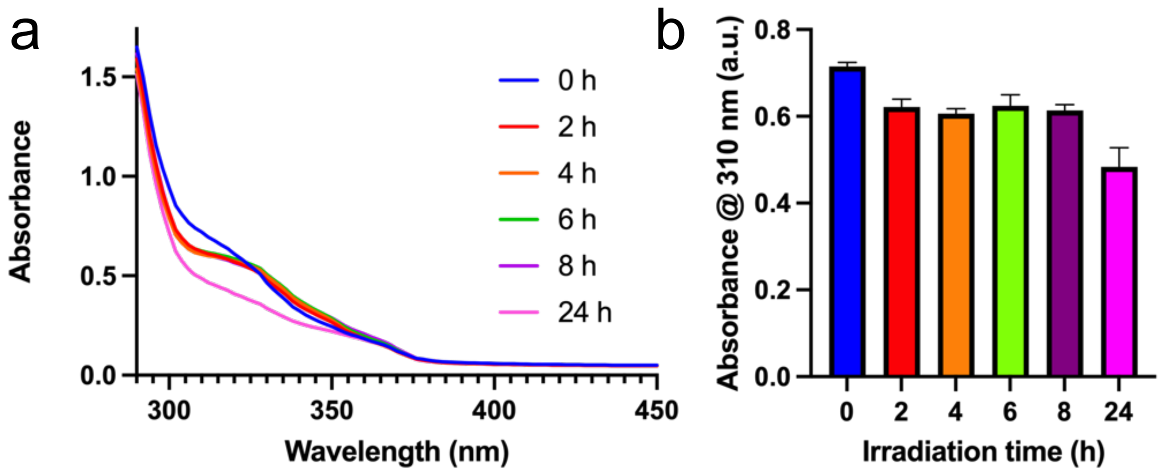

Figure S3. Assessment of chain transfer agent photolysis. UV-Vis absorption spectroscopy of a polymerization conducted in 60 wt% zinc chloride over the course of 24 hours with a) relevant kinetic spectral scans and b) measured absorbance at 310nm. Data displayed as mean ± standard deviation, n=3 per time point.

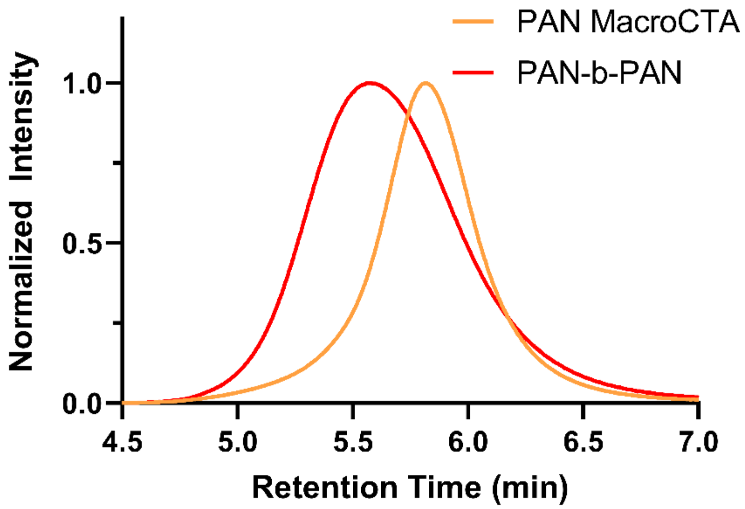

Figure S4. GPC traces of PAN DP 4,000 macroCTA and chain extended PAN-b-PAN DP 8000. PAN DP 4,000 was utilized as a macroCTA for the block copolymerization of PAN-b-PAN in 60 wt% $ZnCl_2$ targeting a total degree of polymerization of 8,000. Acquired GPC traces showing a slight shift toward higher molecular weights while maintaining a narrow dispersity.

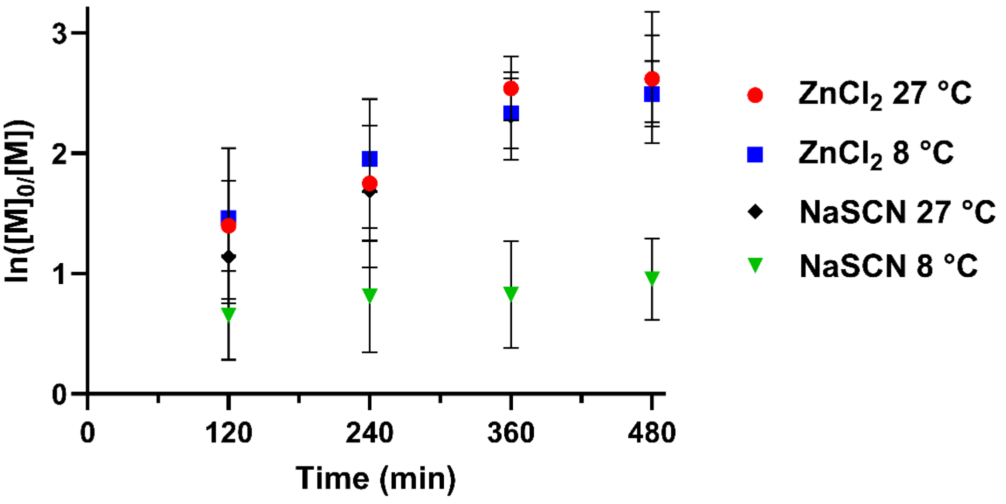

Figure S5. Kinetic plots of aqPI polymerizations conducted at low temperatures. Kinetics of aqPI polymerization conducted at 8 ºC and 27 ºC in aqueous zinc chloride and sodium thiocyanate.

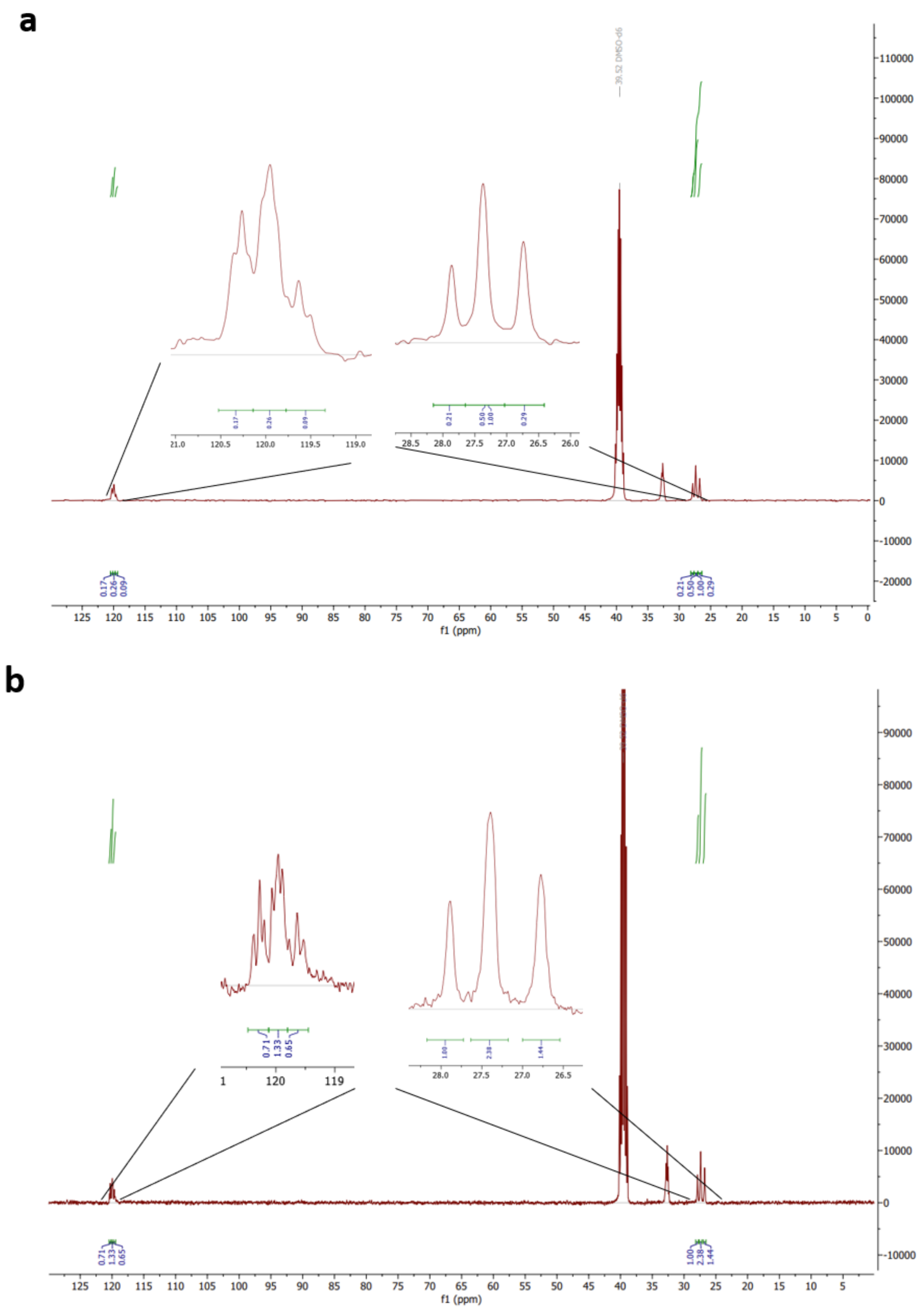

Figure S6. Polymer tacticity investigation by ^{13}C NMR. Representative ^{13}C NMR spectra of polyacrylonitrile DP 500 synthesized in 60 wt% $ZnCl_2$ at a) 27 °C and b) 8 °C.

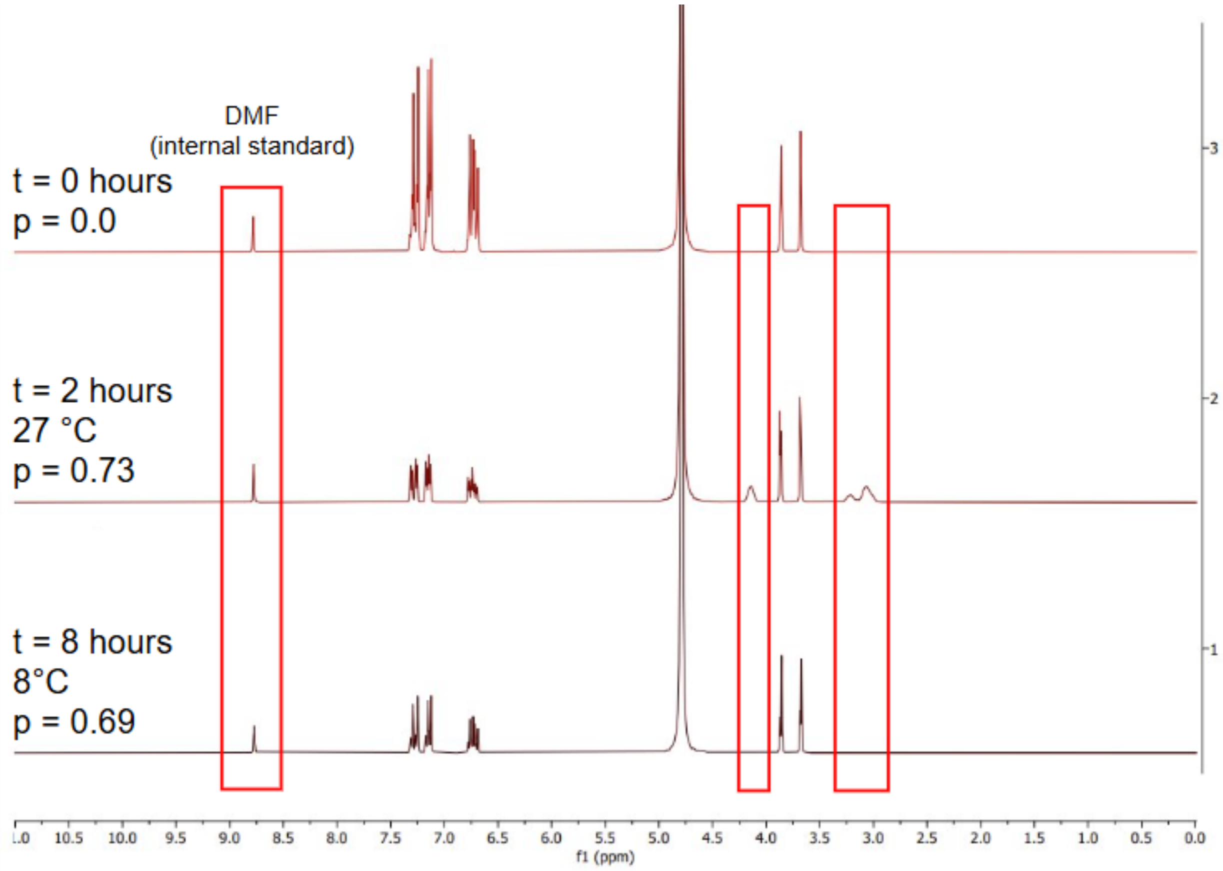

Figure S7. Kinetics study of polyacrylonitrile DP 500 synthesized in 50 wt% NaSCN at 8 and 27 °C. Despite conversion of monomer at earlier stages of reaction, characteristic polymer backbone proton peaks do not appear in samples polymerized at sub-ambient temperatures.

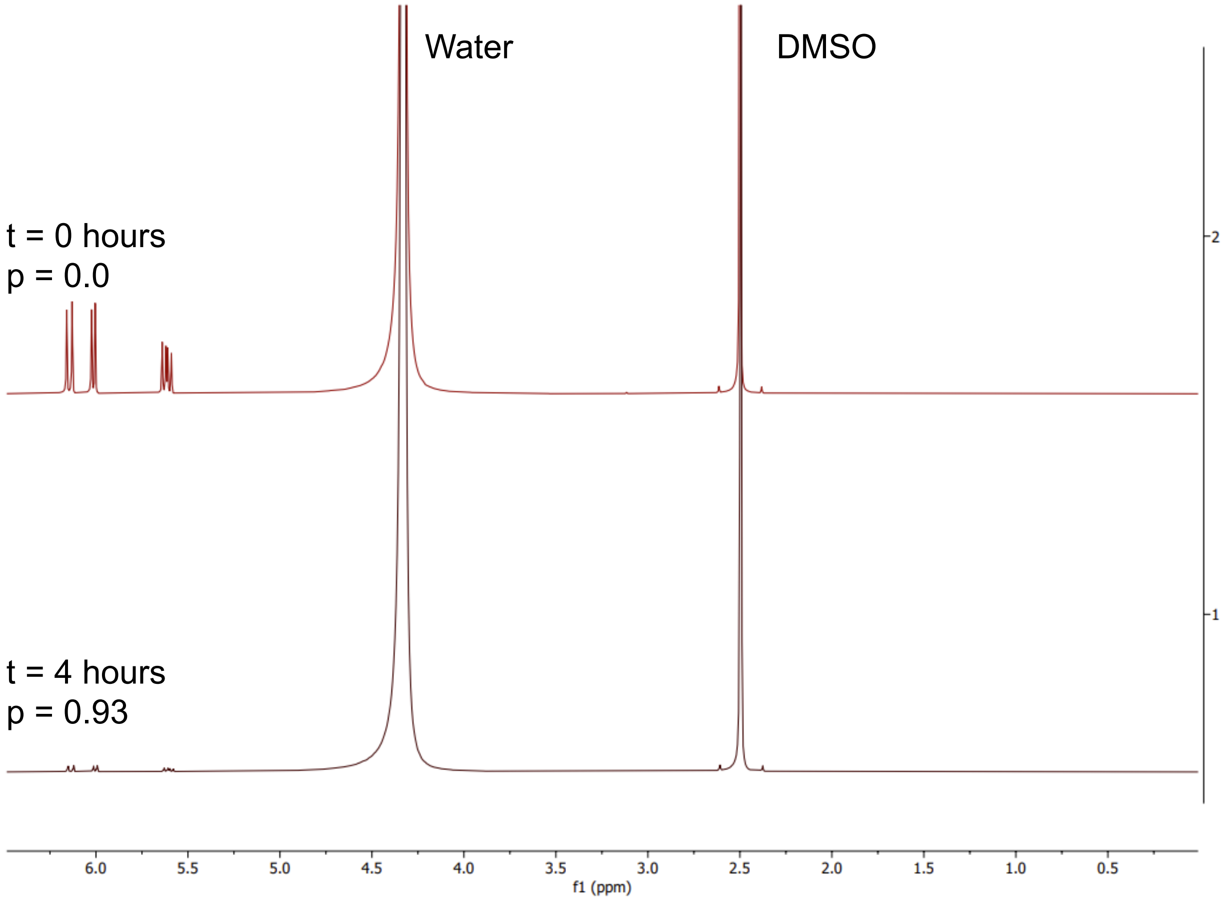

Figure S8. ^{1}H NMR of photo mediated acrylonitrile and thiocyanate interaction. ^{1}H NMR spectra of reaction between sodium thiocyanate and acrylonitrile monomer following 4 hours of ultra-violet light irradiation.

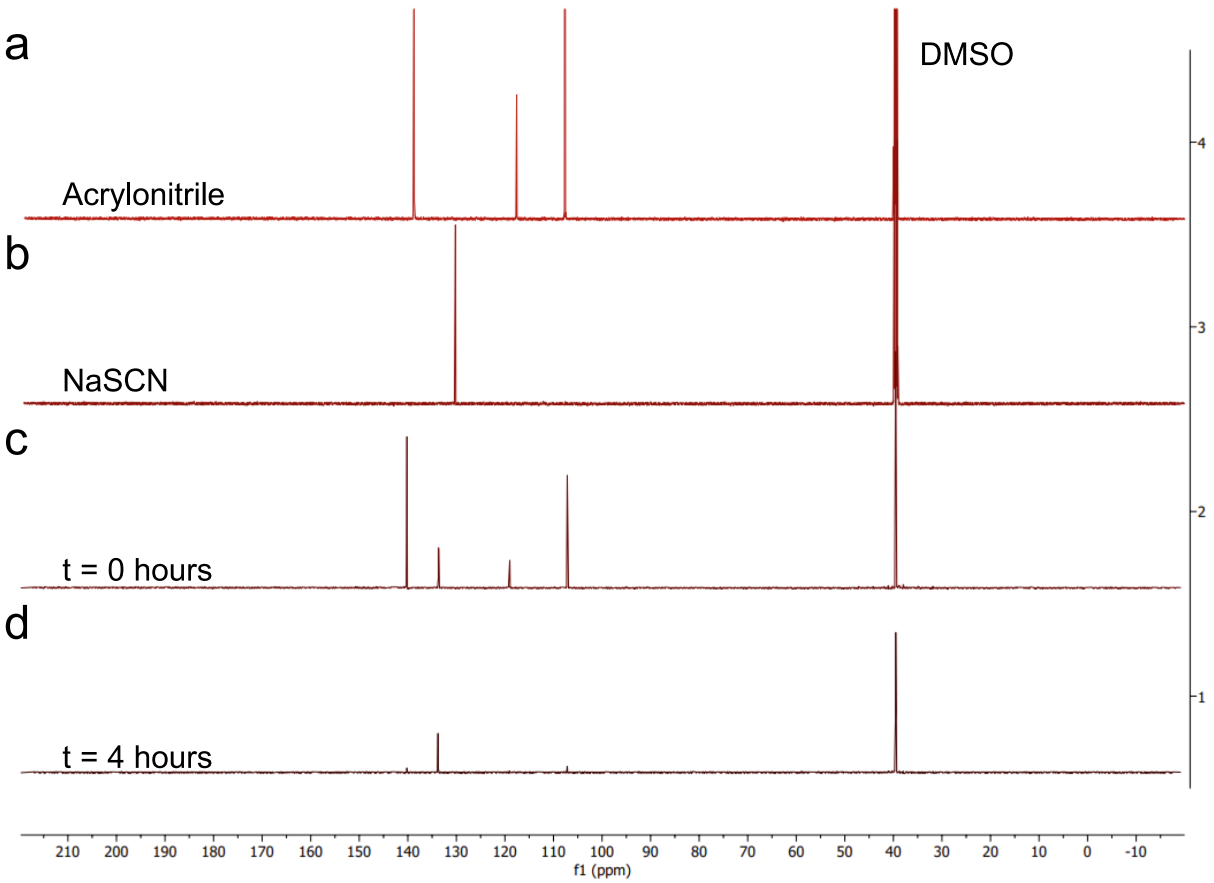

Figure S9. ^{13}C NMR of photo mediated acrylonitrile and thiocyanate interaction. ^{13}C NMR spectra of a) acrylonitrile, b) sodium thiocyanate, c) initial (t = 0 hours) and, d) final (t = 4 hours) timepoints of the reaction.

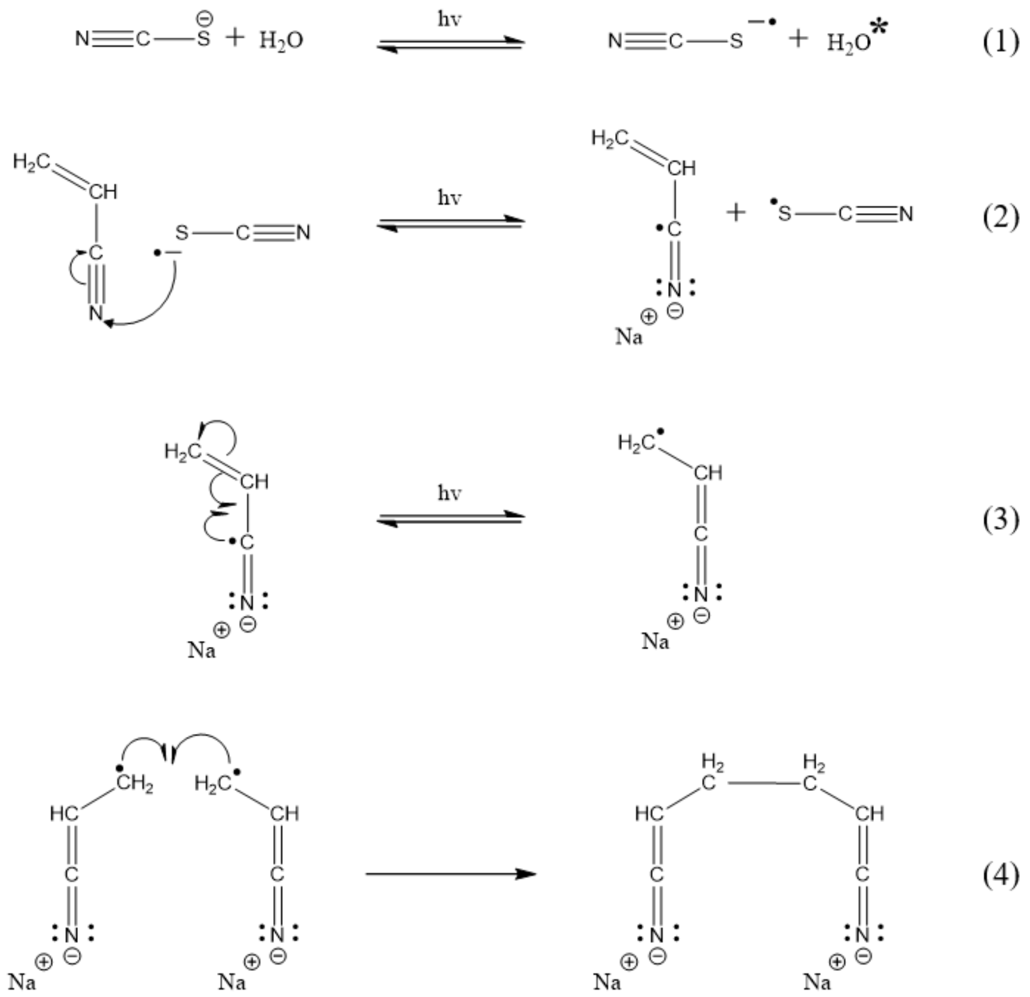

Figure S10. Schematic representation of UV light catalyzed photolysis of aqueous thiocyanate to form radical anions. (1) UV irradiation of aqueous thiocyanate forms a radical anion complex with water. (2) The formation of a weak charge transfer complex between AN and SCN radical anions facilitates electron transfer and carbon-centered radical generation on monomers. (3) Electron transfer induced rearrangement of acrylonitrile monomer is followed by (4) bimolecular coupling of carbon centered radicals resulting in dimers.

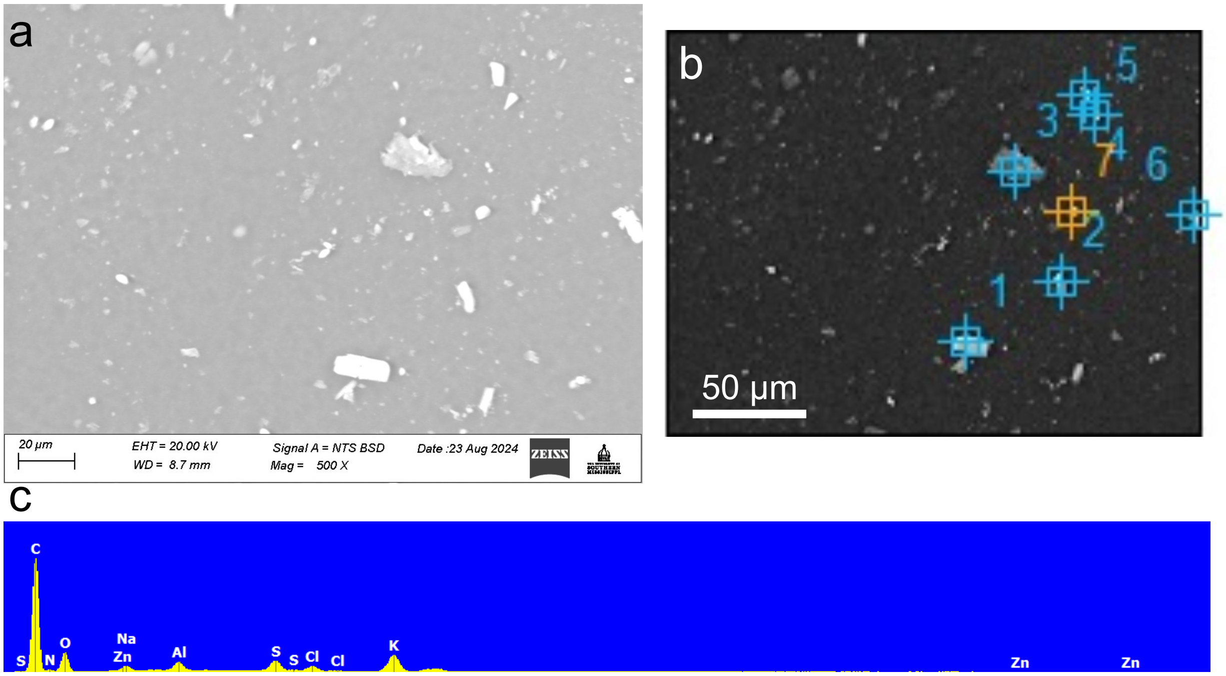

d	C-K (wt %)	N-K (wt %)	O-K (wt %)	Na-K (wt %)	Al-K (wt %)	S-K (wt %)	Cl-K (wt %)	K-K (wt %)	Ca-K (wt %)	Cu-K (wt %)	Zn-K (wt %)
**PAN-washed_pt1**	31.67	7.64	30.38		2.02	0.03		27.83		0.40	0.03
**PAN-washed_pt2**	62.89	9.91	24.13		1.95	0.04		0.80		0.28	0.00
**PAN-washed_pt3**	47.78	8.67	30.79	1.40	1.78	2.42	1.24	5.87			0.04
**PAN-washed_pt4**	61.94	11.20	23.98		1.73	0.03		0.74	0.12	0.26	0.00
**PAN-washed_pt5**	63.11	10.94	22.78		1.97	0.02		0.87		0.31	0.00
**PAN-washed_pt6**	47.94	9.49	33.16		1.74	0.03		7.62			0.02
**PAN-washed_pt7**	62.31	11.24	23.80		1.97	0.05		0.38	0.25		0.00

Figure S11. Scanning electron microscopy (SEM) assessment following polymer purification. a) SEM micrograph of 60 wt% ZnCl2 aqPI synthesized PAN following a single wash and b) spot map used for energy dispersive X-ray spectroscopy (EDS) assessment. c) representative EDS spectrum from spot 3, and d) summary table of weight percentage composition assessed from each point assessed.

4 Supporting Tables

4.1 Table S1. Polymerization kinetics of acrylonitrile DP 2000 with aliquots taken for GPC analysis to confirm agreement between theoretical and experimental M_n.

Sample	Int. std. integration	Monomer peak integration	[M]	M_o/M	$\ln(M_o/M)$	Time (min)	p	$DP_{(th)}$	$Mn_{(th)}$ (g/mol)	$Mn_{(exp)}$ (g/mol)
t0	1	7.51	2.00	1.00	0.00	0	0.00	0	307	-
t1	1	5.39	1.44	1.39	0.33	30	0.28	566	30,300	15,000
t2	1	4.12	1.10	1.82	0.60	75	0.45	905	48,300	26,300
t3	1	3.67	0.98	2.05	0.72	120	0.51	1025	54,700	38,600
t4	1	2.88	0.77	2.61	0.96	240	0.62	1236	65,900	51,100
t5	1	1.89	0.50	3.97	1.38	360	0.75	1500	79,900	57,700
t6	1	1.30	0.35	5.78	1.75	480	0.83	1657	88,300	63,900

5 Supporting Information References

(1) Striegel, A. M. Specific Refractive Index Increment (∂n/∂c) of Polymers at 660 nm and 690 nm. *Chromatographia* **2017**, *80* (6), 989-996. DOI: 10.1007/s10337-017-3294-2.
